# Crystal structure and Hirshfeld surface analysis of (*E*)-3-(2-chloro-6-fluoro­phen­yl)-1-(3-fluoro-4-meth­oxy­phen­yl)prop-2-en-1-one

**DOI:** 10.1107/S2056989016006526

**Published:** 2016-04-22

**Authors:** Nur Hafiq Hanif Hassan, Amzar Ahlami Abdullah, Suhana Arshad, Nuridayanti Che Khalib, Ibrahim Abdul Razak

**Affiliations:** a School of Physics, Universiti Sains Malaysia, 11800 USM, Penang, Malaysia

**Keywords:** crystal structure, chalcone, hydrogen bonding, Hirshfeld surface analysis

## Abstract

In the title chalcone derivative, C_16_H_11_ClF_2_O_2_, the enone group adopts an *E* conformation. The dihedral angle between the benzene rings is 0.47 (9)° and an intra­molecular C—H⋯F hydrogen bond closes an *S*(6) ring. In the crystal, mol­ecules are linked into a three-dimensional network by C—H⋯O hydrogen bonds and aromatic π–π stacking inter­actions are also observed [centroid–centroid separation = 3.5629 (18) Å]. The inter­molecular inter­actions in the crystal structure were qu­anti­fied and analysed using Hirshfeld surface analysis.

## Abstract 2

In the title chalcone derivative, mol­ecules are linked into a three-dimensional network by C—H⋯O hydrogen bonds and aromatic π–π stacking inter­actions are also observed. The inter­molecular inter­actions in the crystal structure were qu­anti­fied and analysed using Hirshfeld surface analysis.

## Chemical context   

Chalcone derivatives possess a wide range of biological properties such as anti­bacterial (Jarag *et al.*, 2011 ▸), anti-inflammatory (Mukherjee *et al.*, 2001 ▸) and anti-oxidant (Arty *et al.*, 2000 ▸) activities. As part of our ongoing studies on chalcone derivatives, we hereby report the synthesis and crystal structure of the title compound, (I).
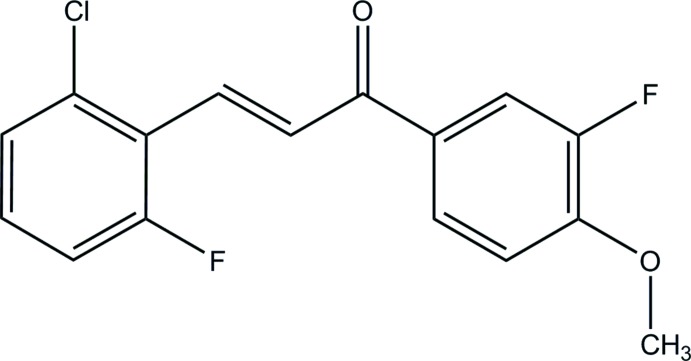


## Structural commentary   

**Figure 1:**
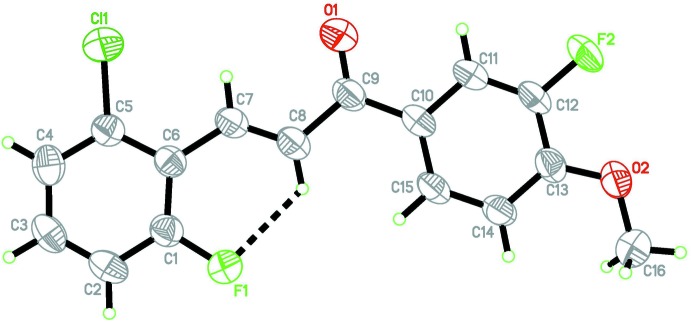
The structure of the title compound, showing 50% probability displacement ellipsoids. The intra­molecular C—H⋯F hydrogen bond is shown as a dashed line.

The mol­ecular structure of (I) is shown in Fig. 1 ▸. The enone moiety (O1/C7–C9) adopts an *E*-conformation with respect to C7=C8 bond. The mol­ecule is slightly twisted at the C9—-C10 bond with a C8—C9—C10—C15 torsion angle of −2.2 (4)° and a maximum deviation of 0.193 (16) Å for atom O1. The dihedral angle between the terminal benzene rings (C1–C6 and C10–C15) is 0.47 (9)°. The least-squares plane through the enone moiety (O1/C7–C9) makes dihedral angles of 2.87 (14) and 3.33 (14)° with the C1–C6 and C10–C15 benzene rings, respectively. An intra­molecular C8—H8*A*⋯F1 hydrogen bond (Table 1 ▸) is observed, generating an *S*(6) ring motif. The bond lengths and angles are comparable with the equivalent data for previously reported structures; (Razak *et al.*, 2009 ▸; Harrison *et al.*, 2006*a* ▸).

## Supra­molecular features   

**Figure 2:**
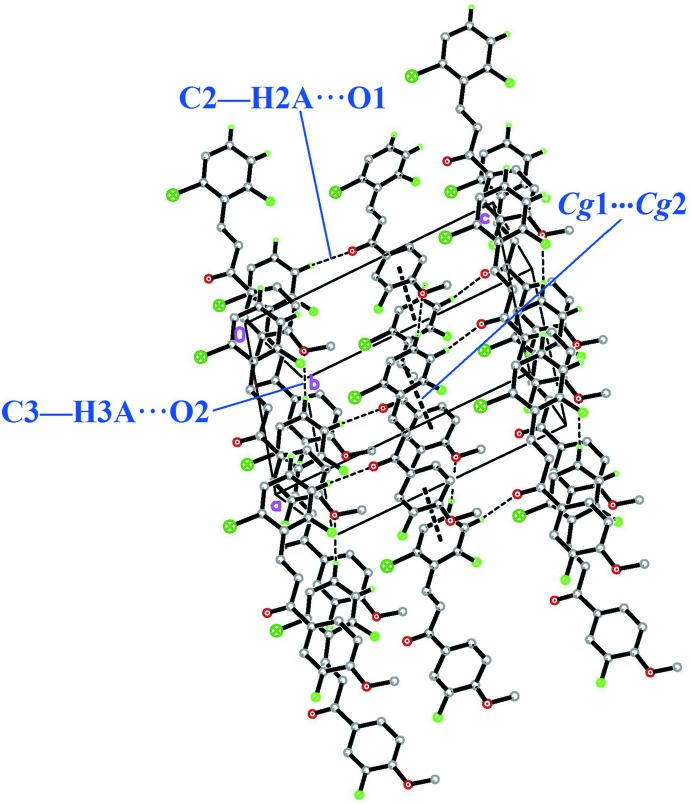
The packing in (I) showing C—H⋯O and π–π inter­actions as dashed lines.

In the crystal, mol­ecules are linked into a three-dimensional network *via* C2—H2*A*⋯O1 (*x* − 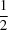, −*y* + 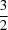, *z* + 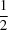) and C3—H3*A*⋯O2 (*x* − 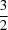, *y* + 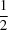, z) hydrogen bonds (Table 1 ▸), as shown in Fig. 2 ▸. The crystal structure also features π–π inter­actions [*Cg*1⋯*Cg*2 (−1 + *x*, *y*, *z*), centroid-to-centroid distance = 3.5629 (18) Å, where *Cg*1 and *Cg*2 are the centroids of the C1–C6 and C10–C15 rings, respectively].

## Analysis of the Hirshfeld Surfaces   

*Crystal Explorer 3.1*(Wolff *et al.*, 2012 ▸) was used to analyse the close contacts in the crystal of (I), which can be summarized with fingerprint plots mapped over *d*_norm_, electrostatic potential, shape index and curvedness. The electrostatic potentials were calculated using *TONTO* (Spackman *et al.*, 2008 ▸; Jayatilaka *et al.*, 2005 ▸) integrated within *Crystal Explorer*. The electrostatic potentials were mapped on Hirshfeld surfaces using the STO-3G basis set at Hartree–Fock level theory over a range ±0.03 au.

**Figure 3:**
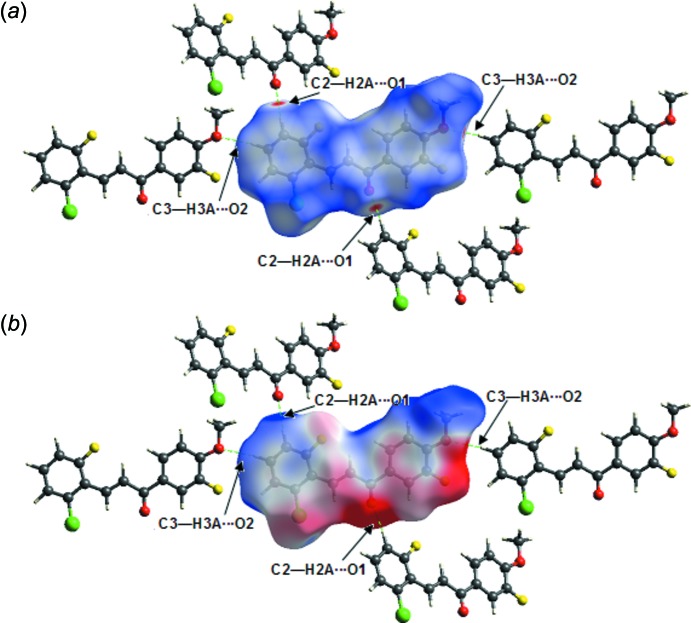
(*a*) *d*_norm_ mapped on Hirshfeld surfaces for visualizing the inter­molecular inter­actions of the title chalcone compound. (*b*) Hirshfeld surfaces mapped over the electrostatic potential. Dotted lines (green) represent hydrogen bonds.

The strong C—H⋯O inter­actions are visualized as bright-red spots between the respective donor and acceptor atoms on the Hirshfeld surfaces mapped over *d*_norm_ (Fig. 3 ▸ *a*) with neighbouring mol­ecules connected by C2—H2*A*⋯O1 and C3—H3*A*⋯O2 hydrogen bonds. This finding is corroborated by Hirshfeld surfaces mapped over the electrostatic potential (Fig. 3 ▸ *b*) showing the negative potential around the oxygen atoms as light-red clouds and the positive potential around hydrogen atoms as light-blue clouds.

**Figure 4:**
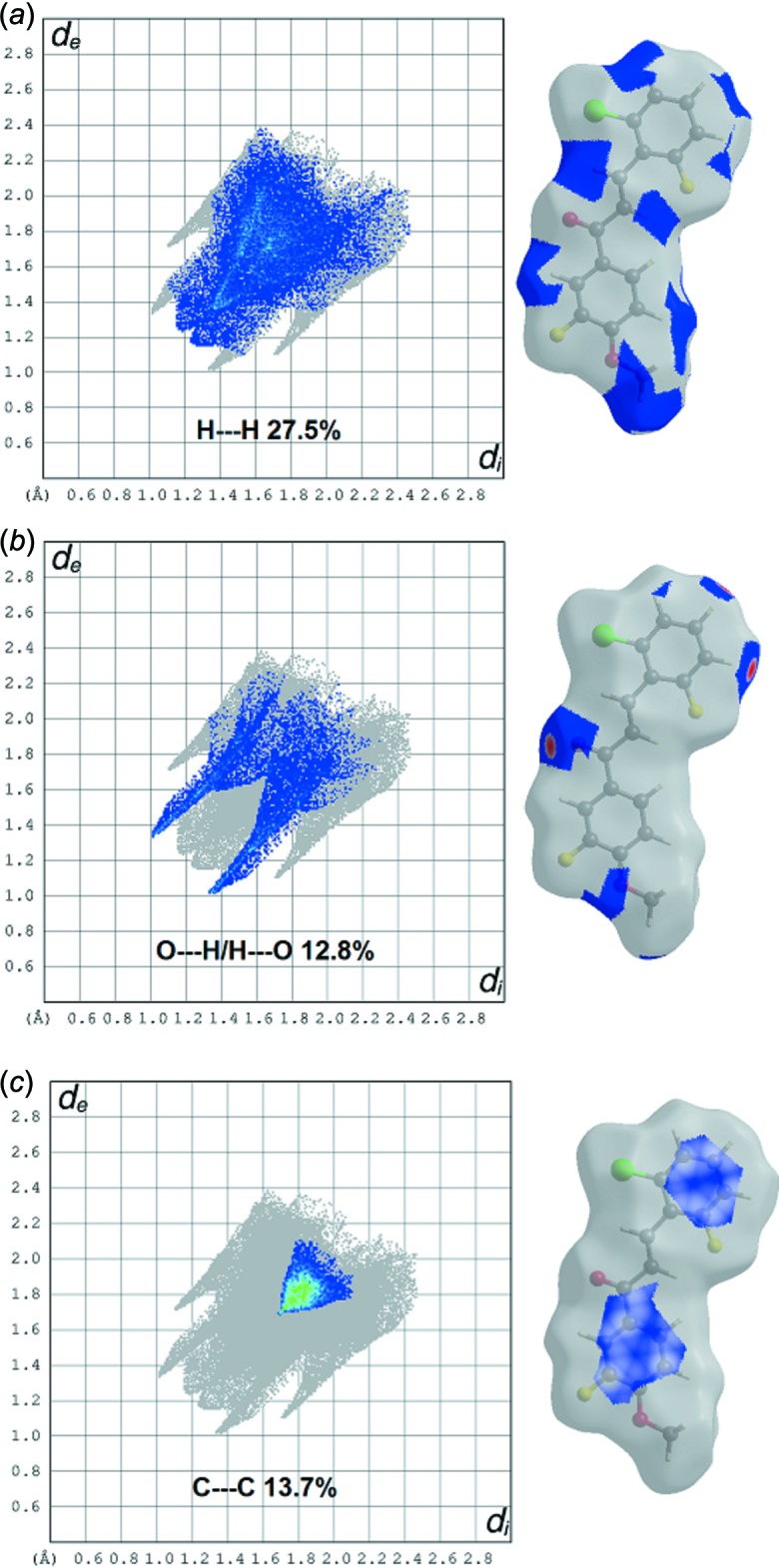
Fingerprint plots for the title chalcone compound, broken down into contributions from specific pairs of atom types. For each plot, the grey shadow is an outline of the complete fingerprint plot. Surfaces to the right highlight the relevant surface patches associated with the specific contacts, with *d*_norm_ mapped in the same manner as Fig. 3 ▸ *a*.

Significant inter­molecular inter­actions are plotted in Fig. 4 ▸: the H⋯H inter­actions appear as the largest region of the fingerprint plot with a high concentration in the middle region, shown in light blue, at *d_e_* = *d_i_* ∼1.4 Å (Fig. 4 ▸ *a*) with overall Hirshfeld surfaces of 27.5%. The contribution from the O⋯H/H⋯O contacts, corresponding to C—H⋯O inter­actions, is represented by a pair of sharp spikes characteristic of a strong hydrogen-bond inter­action having almost the same *d_e_* + *d_i_* ∼2.3 Å (Fig. 4 ▸ *b*).

**Figure 5:**
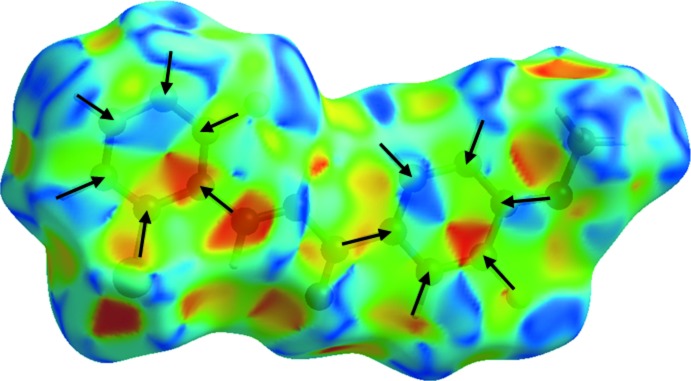
Hirshfeld surfaces mapped over the shape index of the title chalcone compound.

**Figure 6:**
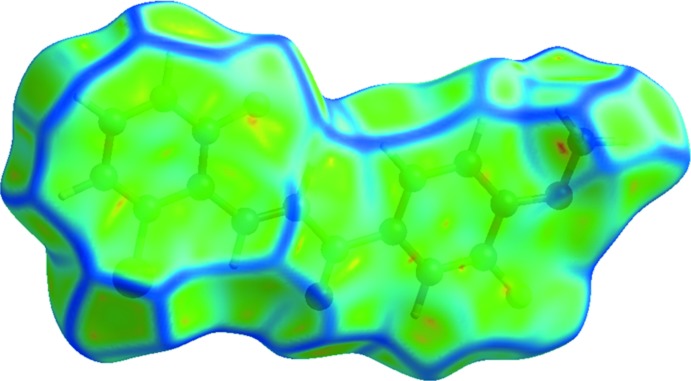
Hirshfeld surfaces mapped over curvedness of the title chalcone compound.

The C⋯C contacts, which refer to π–·π stacking inter­actions, contribute 13.7% of the Hirshfeld surfaces. This appears as a distinct triangle at around *d_e_* = *d_i_* ∼1.8 Å (Fig. 4 ▸ *c*). The presence of the π–π stacking inter­actions is also indicated by the appearance of red and blue triangles on the shape-indexed surfaces, identified with black arrows in Fig. 5 ▸, and in the flat regions on the Hirshfeld surfaces mapped over curvedness in Fig. 6 ▸.

## Synthesis and crystallization   

A mixture of 3-fluoro-4-meth­oxy­aceto­phenone (0.1 mol, 0.08 g) and 2-chloro-6-fluoro­benzaldehyde (0.1 mol, 0.08 g) was dissolved in methanol (20 ml). A catalytic amount of NaOH (5 ml, 20%) was added to the solution dropwise with vigorous stirring. The reaction mixture was stirred for about 5–6 h at room temperature. After stirring, the contents of the flask were poured into ice-cold water (50 ml) and the resulting crude solid was collected by filtration. Brownish blocks of (I) were grown from an acetone solution by slow evaporation.

## Refinement details   

Crystal data collection and structure refinement details are summarized in Table 2 ▸. All H atoms were positioned geometrically (C—H = 0.93 Å) and refined using a riding model with *U*_iso_(H) = 1.2*U*_eq_(C). In the final refinement, the most disagreeable reflection (020) was omitted.

## Supplementary Material

Crystal structure: contains datablock(s) I. DOI: 10.1107/S2056989016006526/hb7578sup1.cif

Structure factors: contains datablock(s) I. DOI: 10.1107/S2056989016006526/hb7578Isup2.hkl

Supporting information file. DOI: 10.1107/S2056989016006526/hb7578Isup3.cml

CCDC reference: 1474605

Additional supporting information:  crystallographic information; 3D view; checkCIF report

## Figures and Tables

**Table 1:** Hydrogen-bond geometry (Å, °)

*D*—H⋯*A*	*D*—H	H⋯*A*	*D*⋯*A*	*D*—H⋯*A*
C2—H2*A*⋯O1^i^	0.93	2.50	3.391 (4)	162
C3—H3*A*⋯O2^ii^	0.93	2.52	3.441 (4)	171
C8—H8*A*⋯F1	0.93	2.21	2.842 (4)	124

Symmetry codes: (i) 
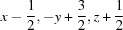
; (ii) 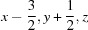.

**Table 2:** Experimental details

Crystal data	
Chemical formula	C_16_H_11_ClF_2_O_2_
*M*_r_	308.70
Crystal system, space group	Monoclinic, *C* *c*
Temperature (K)	294
*a*, *b*, *c* (Å)	9.0832 (13), 11.1072 (13), 13.9564 (17)
β (°)	102.027 (3)
*V* (Å^3^)	1377.1 (3)
*Z*	4
Radiation type	Mo *K*α
μ (mm^−1^)	0.30
Crystal size (mm)	0.45 × 0.17 × 0.13

Data collection	
Diffractometer	Bruker *SMART* APEXII CCD
Absorption correction	Multi-scan (*SADABS*; Bruker, 2009 ▸)
*T*_min_, *T*_max_	0.791, 0.889
No. of measured, independent and observed [*I* > 2σ(*I*)] reflections	14473, 4003, 3111
*R*_int_	0.031
(sin θ/λ)_max_ (Å^−1^)	0.705

Refinement	
*R*[*F* ^2^ > 2σ(*F* ^2^)], *wR*(*F* ^2^), *S*	0.039, 0.116, 1.05
No. of reflections	4003
No. of parameters	191
No. of restraints	2
H-atom treatment	H-atom parameters constrained
Δρ_max_, Δρ_min_ (e Å^−3^)	0.20, −0.27
Absolute structure	Flack *x* determined using 1298 quotients [(*I* ^+^)−(*I* ^−^)]/[(*I* ^+^)+(*I* ^−^)] Parsons *et al.* (2013 ▸)
Absolute structure parameter	0.08 (2)

Computer programs: *APEX2* and *SAINT* (Bruker, 2009 ▸), *SHELXS97* and *SHELXTL* (Sheldrick 2008 ▸), *SHELXL2014* (Sheldrick, 2015 ▸) and *PLATON* (Spek, 2009 ▸).

## supplementary crystallographic information

### Crystal data

**Table:**

C_16_H_11_ClF_2_O_2_	*F*(000) = 632
*M**_r_* = 308.70	*D*_x_ = 1.489 Mg m^−^^3^
Monoclinic, *C**c*	Mo *K*α radiation, λ = 0.71073 Å
*a* = 9.0832 (13) Å	Cell parameters from 4692 reflections
*b* = 11.1072 (13) Å	θ = 2.9–28.9°
*c* = 13.9564 (17) Å	µ = 0.30 mm^−^^1^
β = 102.027 (3)°	*T* = 294 K
*V* = 1377.1 (3) Å^3^	Block, brown
*Z* = 4	0.45 × 0.17 × 0.13 mm

### Data collection

**Table:**

Bruker SMART APEXII CCD diffractometer	4003 independent reflections
Radiation source: fine-focus sealed tube	3111 reflections with *I* > 2σ(*I*)
Graphite monochromator	*R*_int_ = 0.031
φ and ω scans	θ_max_ = 30.1°, θ_min_ = 2.9°
Absorption correction: multi-scan (*SADABS*; Bruker, 2009)	*h* = −12→12
*T*_min_ = 0.791, *T*_max_ = 0.889	*k* = −15→15
14473 measured reflections	*l* = −19→19

### Refinement

**Table:**

Refinement on *F*^2^	Hydrogen site location: inferred from neighbouring sites
Least-squares matrix: full	H-atom parameters constrained
*R*[*F*^2^ > 2σ(*F*^2^)] = 0.039	*w* = 1/[σ^2^(*F*_o_^2^) + (0.0664*P*)^2^ + 0.0639*P*] where *P* = (*F*_o_^2^ + 2*F*_c_^2^)/3
*wR*(*F*^2^) = 0.116	(Δ/σ)_max_ < 0.001
*S* = 1.05	Δρ_max_ = 0.20 e Å^−^^3^
4003 reflections	Δρ_min_ = −0.27 e Å^−^^3^
191 parameters	Absolute structure: Flack *x* determined using 1298 quotients [(*I*^+^)-(*I*^-^)]/[(*I*^+^)+(*I*^-^)] Parsons *et al.* (2013)
2 restraints	Absolute structure parameter: 0.08 (2)

### Special details

**Table:**

Geometry. All esds (except the esd in the dihedral angle between two l.s. planes) are estimated using the full covariance matrix. The cell esds are taken into account individually in the estimation of esds in distances, angles and torsion angles; correlations between esds in cell parameters are only used when they are defined by crystal symmetry. An approximate (isotropic) treatment of cell esds is used for estimating esds involving l.s. planes.

### Fractional atomic coordinates and isotropic or equivalent isotropic displacement parameters (Å^2^)

**Table:**

	*x*	*y*	*z*	*U*_iso_*/*U*_eq_	
Cl1	0.01382 (11)	0.90043 (10)	0.28908 (7)	0.0766 (3)	
F1	0.2567 (2)	0.7732 (2)	0.63725 (14)	0.0679 (6)	
F2	1.0123 (2)	0.5464 (2)	0.42338 (15)	0.0676 (5)	
O1	0.5069 (4)	0.7363 (3)	0.3366 (2)	0.0864 (9)	
O2	1.0692 (2)	0.5000 (2)	0.61111 (18)	0.0654 (6)	
C1	0.1380 (3)	0.8232 (3)	0.5705 (2)	0.0473 (6)	
C2	0.0177 (4)	0.8618 (3)	0.6081 (2)	0.0558 (7)	
H2A	0.0181	0.8538	0.6745	0.067*	
C3	−0.1020 (3)	0.9121 (3)	0.5458 (3)	0.0555 (7)	
H3A	−0.1844	0.9391	0.5698	0.067*	
C4	−0.1017 (3)	0.9231 (3)	0.4480 (3)	0.0533 (7)	
H4A	−0.1837	0.9573	0.4057	0.064*	
C5	0.0209 (3)	0.8832 (2)	0.4124 (2)	0.0447 (6)	
C6	0.1479 (3)	0.8313 (2)	0.47289 (19)	0.0409 (5)	
C7	0.2772 (3)	0.7932 (3)	0.4327 (2)	0.0480 (6)	
H7A	0.2670	0.8034	0.3655	0.058*	
C8	0.4052 (4)	0.7467 (3)	0.4783 (2)	0.0527 (6)	
H8A	0.4215	0.7321	0.5453	0.063*	
C9	0.5248 (3)	0.7170 (3)	0.4236 (2)	0.0501 (6)	
C10	0.6672 (3)	0.6632 (2)	0.4787 (2)	0.0428 (6)	
C11	0.7762 (3)	0.6300 (3)	0.4255 (2)	0.0458 (6)	
H11A	0.7598	0.6433	0.3583	0.055*	
C12	0.9060 (3)	0.5780 (2)	0.4739 (2)	0.0463 (6)	
C13	0.9367 (3)	0.5544 (3)	0.5741 (2)	0.0478 (6)	
C14	0.8298 (3)	0.5889 (3)	0.6265 (2)	0.0505 (6)	
H14A	0.8471	0.5762	0.6938	0.061*	
C15	0.6969 (3)	0.6426 (3)	0.5783 (2)	0.0480 (6)	
H15A	0.6259	0.6653	0.6142	0.058*	
C16	1.1014 (5)	0.4726 (5)	0.7133 (3)	0.0857 (13)	
H16A	1.1972	0.4329	0.7304	0.129*	
H16B	1.0245	0.4206	0.7278	0.129*	
H16C	1.1041	0.5457	0.7502	0.129*	

### Atomic displacement parameters (Å^2^)

**Table:**

	*U*^11^	*U*^22^	*U*^33^	*U*^12^	*U*^13^	*U*^23^
Cl1	0.0769 (5)	0.1065 (7)	0.0450 (4)	0.0182 (5)	0.0090 (3)	0.0033 (4)
F1	0.0590 (11)	0.0974 (14)	0.0482 (10)	0.0220 (9)	0.0137 (8)	0.0121 (10)
F2	0.0564 (9)	0.0931 (14)	0.0632 (11)	0.0103 (9)	0.0350 (9)	−0.0092 (10)
O1	0.0880 (18)	0.125 (2)	0.0543 (14)	0.0458 (17)	0.0320 (13)	0.0164 (15)
O2	0.0398 (10)	0.1009 (18)	0.0566 (13)	0.0060 (11)	0.0124 (9)	−0.0102 (12)
C1	0.0477 (13)	0.0491 (14)	0.0478 (14)	0.0008 (11)	0.0161 (11)	0.0033 (12)
C2	0.0601 (17)	0.0623 (17)	0.0529 (16)	−0.0056 (14)	0.0297 (14)	−0.0028 (14)
C3	0.0464 (14)	0.0587 (16)	0.0685 (19)	−0.0037 (12)	0.0281 (13)	−0.0069 (14)
C4	0.0398 (13)	0.0518 (15)	0.068 (2)	−0.0015 (11)	0.0099 (12)	−0.0036 (14)
C5	0.0441 (13)	0.0465 (14)	0.0436 (13)	−0.0029 (11)	0.0098 (11)	−0.0027 (10)
C6	0.0421 (12)	0.0388 (12)	0.0435 (13)	−0.0024 (9)	0.0130 (10)	−0.0011 (10)
C7	0.0517 (14)	0.0520 (15)	0.0441 (14)	0.0054 (12)	0.0189 (11)	0.0010 (11)
C8	0.0542 (15)	0.0589 (16)	0.0501 (15)	0.0084 (13)	0.0225 (12)	−0.0005 (13)
C9	0.0540 (14)	0.0515 (14)	0.0510 (15)	0.0076 (12)	0.0247 (12)	−0.0003 (12)
C10	0.0471 (13)	0.0385 (12)	0.0490 (14)	−0.0052 (10)	0.0242 (11)	−0.0053 (10)
C11	0.0503 (14)	0.0491 (14)	0.0437 (13)	−0.0043 (11)	0.0229 (11)	−0.0057 (11)
C12	0.0426 (12)	0.0540 (15)	0.0491 (14)	−0.0051 (11)	0.0246 (11)	−0.0112 (12)
C13	0.0365 (12)	0.0564 (15)	0.0527 (15)	−0.0066 (11)	0.0147 (11)	−0.0110 (12)
C14	0.0449 (13)	0.0684 (18)	0.0418 (14)	−0.0043 (12)	0.0174 (11)	−0.0068 (13)
C15	0.0446 (12)	0.0583 (15)	0.0473 (14)	−0.0024 (11)	0.0236 (11)	−0.0070 (12)
C16	0.0552 (19)	0.142 (4)	0.057 (2)	0.014 (2)	0.0050 (16)	−0.002 (2)

### Geometric parameters (Å, º)

**Table:**

Cl1—C5	1.720 (3)	C7—H7A	0.9300
F1—C1	1.386 (3)	C8—C9	1.489 (4)
F2—C12	1.354 (3)	C8—H8A	0.9300
O1—C9	1.209 (4)	C9—C10	1.485 (4)
O2—C13	1.349 (4)	C10—C15	1.379 (4)
O2—C16	1.427 (5)	C10—C11	1.405 (3)
C1—C2	1.376 (4)	C11—C12	1.359 (4)
C1—C6	1.387 (4)	C11—H11A	0.9300
C2—C3	1.363 (5)	C12—C13	1.393 (4)
C2—H2A	0.9300	C13—C14	1.386 (4)
C3—C4	1.371 (5)	C14—C15	1.389 (4)
C3—H3A	0.9300	C14—H14A	0.9300
C4—C5	1.383 (4)	C15—H15A	0.9300
C4—H4A	0.9300	C16—H16A	0.9600
C5—C6	1.403 (4)	C16—H16B	0.9600
C6—C7	1.466 (3)	C16—H16C	0.9600
C7—C8	1.309 (4)		
			
C13—O2—C16	117.3 (3)	O1—C9—C8	121.0 (3)
C2—C1—F1	115.9 (3)	C10—C9—C8	118.2 (3)
C2—C1—C6	125.0 (3)	C15—C10—C11	118.5 (3)
F1—C1—C6	119.1 (2)	C15—C10—C9	123.7 (2)
C3—C2—C1	118.4 (3)	C11—C10—C9	117.7 (2)
C3—C2—H2A	120.8	C12—C11—C10	118.9 (3)
C1—C2—H2A	120.8	C12—C11—H11A	120.6
C2—C3—C4	120.3 (3)	C10—C11—H11A	120.6
C2—C3—H3A	119.8	F2—C12—C11	119.4 (3)
C4—C3—H3A	119.8	F2—C12—C13	117.3 (3)
C3—C4—C5	119.9 (3)	C11—C12—C13	123.3 (2)
C3—C4—H4A	120.1	O2—C13—C14	126.0 (3)
C5—C4—H4A	120.1	O2—C13—C12	116.4 (2)
C4—C5—C6	122.5 (3)	C14—C13—C12	117.6 (3)
C4—C5—Cl1	117.3 (2)	C13—C14—C15	119.8 (3)
C6—C5—Cl1	120.1 (2)	C13—C14—H14A	120.1
C1—C6—C5	113.8 (2)	C15—C14—H14A	120.1
C1—C6—C7	125.3 (3)	C10—C15—C14	121.9 (2)
C5—C6—C7	120.9 (2)	C10—C15—H15A	119.1
C8—C7—C6	129.1 (3)	C14—C15—H15A	119.1
C8—C7—H7A	115.5	O2—C16—H16A	109.5
C6—C7—H7A	115.5	O2—C16—H16B	109.5
C7—C8—C9	120.5 (3)	H16A—C16—H16B	109.5
C7—C8—H8A	119.7	O2—C16—H16C	109.5
C9—C8—H8A	119.7	H16A—C16—H16C	109.5
O1—C9—C10	120.8 (3)	H16B—C16—H16C	109.5
			
F1—C1—C2—C3	179.8 (3)	O1—C9—C10—C15	177.8 (3)
C6—C1—C2—C3	0.1 (5)	C8—C9—C10—C15	−2.2 (4)
C1—C2—C3—C4	0.2 (5)	O1—C9—C10—C11	−3.2 (4)
C2—C3—C4—C5	−0.1 (5)	C8—C9—C10—C11	176.9 (3)
C3—C4—C5—C6	−0.3 (4)	C15—C10—C11—C12	0.5 (4)
C3—C4—C5—Cl1	−179.8 (2)	C9—C10—C11—C12	−178.5 (2)
C2—C1—C6—C5	−0.4 (4)	C10—C11—C12—F2	−179.6 (2)
F1—C1—C6—C5	179.9 (2)	C10—C11—C12—C13	0.7 (4)
C2—C1—C6—C7	178.4 (3)	C16—O2—C13—C14	1.7 (5)
F1—C1—C6—C7	−1.3 (4)	C16—O2—C13—C12	−178.6 (3)
C4—C5—C6—C1	0.5 (4)	F2—C12—C13—O2	−1.1 (4)
Cl1—C5—C6—C1	180.0 (2)	C11—C12—C13—O2	178.6 (3)
C4—C5—C6—C7	−178.3 (3)	F2—C12—C13—C14	178.7 (2)
Cl1—C5—C6—C7	1.2 (3)	C11—C12—C13—C14	−1.7 (4)
C1—C6—C7—C8	−0.7 (5)	O2—C13—C14—C15	−179.0 (3)
C5—C6—C7—C8	178.0 (3)	C12—C13—C14—C15	1.3 (4)
C6—C7—C8—C9	−178.2 (3)	C11—C10—C15—C14	−0.9 (4)
C7—C8—C9—O1	0.8 (5)	C9—C10—C15—C14	178.2 (3)
C7—C8—C9—C10	−179.3 (3)	C13—C14—C15—C10	−0.1 (4)

### Hydrogen-bond geometry (Å, º)

**Table:**

*D*—H···*A*	*D*—H	H···*A*	*D*···*A*	*D*—H···*A*
C2—H2*A*···O1^i^	0.93	2.50	3.391 (4)	162
C3—H3*A*···O2^ii^	0.93	2.52	3.441 (4)	171
C8—H8*A*···F1	0.93	2.21	2.842 (4)	124

Symmetry codes: (i) *x*−1/2, −*y*+3/2, *z*+1/2; (ii) *x*−3/2, *y*+1/2, *z*.
